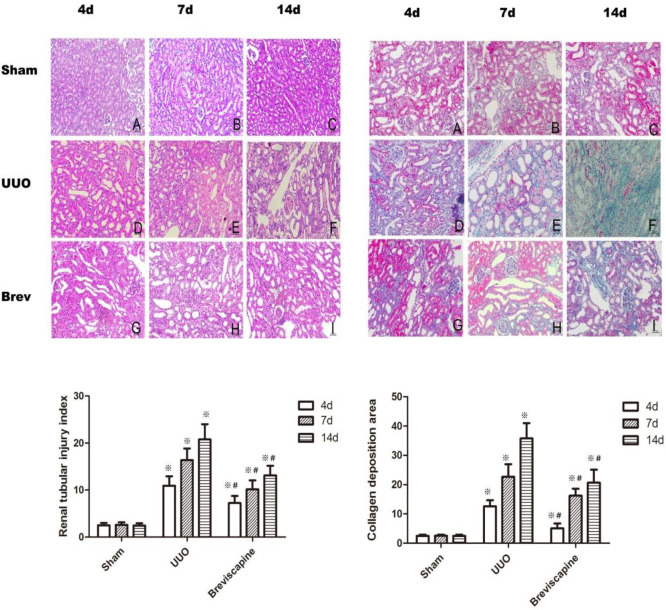#  Erratum: Breviscapine prevents downregulation of renal water and sodium transport proteins in response to unilateral ureteral obstruction

**Published:** 2022-02

**Authors:** Yang Mei, Zhuang Yangyang, Luo Shuai, Jin Hao, Yang Yirong, Cai Yong, Xia Peng, Chen Bicheng, Zhang Yan

**Affiliations:** 1 Department of Intensive Care Unit, The First Affiliate Hospital of Wenzhou Medicine University, China; 2 Department of Transplantation Center, the First Affiliate Hospital of Wenzhou Medicine University, China; 3 Key Laboratory of Surgery, Department of Surgery, The First Affiliated Hospital of Wenzhou Medicine University, China

 The original article entitled “Breviscapine prevents downregulation of renal water and sodium transport proteins in response to unilateral ureteral obstruction” corresponded by Zhang Yan was published with incorrect pictures.

Due to author request the pictures below should replace the ones in Figure 1 in the original article.

**Figure:**